# Discovery tools: How powerful new scientific methods and instruments emerge and catalyze innovation

**DOI:** 10.1093/pnasnexus/pgag107

**Published:** 2026-04-09

**Authors:** Alexander Krauss

**Affiliations:** Spanish National Research Council, 08193 Barcelona, Spain; London School of Economics, Houghton Street, London WC2A 2AE, United Kingdom

**Keywords:** scientific tools, scientific methods, method innovation, tool-driven discovery, methods labs

## Abstract

Powerful new methods and tools drive scientific progress—but how do we actually make such innovations? No theory yet explains how we invent major tools across fields. To address this gap, we examine all nobel–prize–winning method discoveries—that enabled breakthrough findings not possible without them—and we trace science's most influential toolmakers across fields, from Ernst Ruska's electron microscope and Kary Mullis's PCR method to Ernest Lawrence's particle accelerator. Here, we lay out the critical pathways taken to create these groundbreaking tools. We introduce a taxonomy of science's methods and tools: a scientific table of discovery methods that is a map of underexplored and unexplored method opportunities. By mapping the method landscape, we reveal gaps and possibilities that help guide where methods can be adapted, recombined, or strategically developed to catalyze discovery. It can help identify and predict untapped combinations of tools—and where the next breakthroughs can come from. What if we no longer wait for new discovery tools to emerge by chance but begin deliberately prioritizing their development? How many big breakthroughs are we missing because we have not yet strategically focused on designing the needed tools? We also outline the need for establishing methods labs and hubs—as incubators of innovation—that catalyze tool creation.

## Introduction

“The important thing in science is not so much to obtain new facts as to discover new ways of thinking about them,” as the physicist Lawrence Bragg noted. And the biochemist Albert Szent-Gyorgyi highlighted that “Discovery consists of seeing what everybody has seen and thinking what nobody has thought.” Yet Bragg's nobel-winning discovery of the structure of crystals was made possible by developing a new X-ray spectrometer (1). Szent-Gyorgyi's nobel-winning discovery of isolating vitamin C was triggered by applying recently created assay techniques and the ultracentrifuge.

Here, we move beyond individual anecdotes of a discovery to systematically study the powerful tools and methods used to trigger science's major discoveries. By building microscopes and telescopes, we have uncovered a world of microorganisms, nanoparticles, molecules, and galaxies, reshaping our understanding of both life and the universe (2, 3). Through advances in statistics and computing, we can process vast data about nearly everything in science, reveal previously hidden patterns and model complex systems—from neural networks to the Earth's climate—that are otherwise too vast to study (4, 5). By inventing X-ray devices, MRI, and computed tomography scans, we can create extraordinarily precise images inside the human body (6, 7). Through machine learning methods, we can analyze massive datasets, run experiments, and generate predictions by spotting patterns at exceptional speed (8, 9). Such remarkable tools often uncover what we did not even know existed. Every major scientific breakthrough is, at its core, a breakthrough in method—a new way to observe, measure, and understand the world. But despite their vast impact, we still have not yet answered crucial questions: How do we actually develop such powerful tools of discovery? How can we upgrade them faster? How can we better use them to their fullest potential?

Existing research studying scientific methods has focused largely on three areas. First, researchers have analyzed a specific method—like statistical techniques (4, 5), microscopy approaches (2, 10), or lasers (11). Second, meta-researchers have examined methodological questions about science itself, including reproducibility, peer review, and assessing research impact (12–14). Third, philosophers have explored scientific methodology from a conceptual perspective, investigating principles like the falsifiability of theories or the classic scientific method of hypothesis testing (15–17). Yet no systematic studies—applied or theoretical—explain how science's diverse methods and tools, from cutting-edge telescopes to supercomputers, emerge across fields and how we can speed up their development. This gap in the empirical, meta-research, and philosophical literature is striking given the increasing complexity and interdisciplinarity of modern science, where breakthroughs depend on novel instruments and techniques. The lack of a systematic understanding also has practical consequences for science policy, funding, and the organization of research.

Here, we address this gap by identifying the patterns that underlie methodological innovation and evolution. We analyze all nobel–prize–winning method discoveries (18) and trace science's most influential toolmakers across fields to explain how leading scientific methods and tools are developed (4, 6, 19–26 ). The aim is to contribute to a more comprehensive theory of scientific change—one that accounts not only for ideas and theories but also for the tools that make them possible. Understanding these patterns can help design ways to accelerate methodological innovations across disciplinary boundaries and help predict what techniques are likely to have broad impact.

After examining how science's leading tools are developed, we outline a taxonomy of discovery tools: a scientific table of methods that maps underused and unused method opportunities. This conceptual framework helps guide where methods can be transferred, recombined, or strategically created to spur discovery. Building on this analysis, we also propose the creation of global methods labs and hubs dedicated to accelerating tool innovation—and outline what these labs can look like. We finally lay out the practical steps and process we can take to design and innovate tools. We need to shift attention from “what” we discover to “how” we discover and how we can discover faster. Together, the insights offer a roadmap for speeding up scientific progress through powerful new tools.

Understanding the dynamics of methodological innovation is essential because tools not only enable us to perceive, measure, and experiment in ways we never imagined but also think in ways unimaginable without them. They make discovery more strategic and less driven by chance. They accelerate the pace and scale of discovery—for example, through new technologies like high-powered computing, machine learning, and data analytics. And tools make science precise and reproducible. Ultimately, if researchers become aware of the methodological limits, challenges, and opportunities of their research, they are better able to push those limits, tackle those challenges, and take advantage of those opportunities. They are better able to design, experiment with, and leverage new tools that often redefine what we can discover.

## Measuring scientific methods and tools

We collect data on all 533 nobel–prize–winning discoveries across science—from the first year of the prize in 1901 to 2022 (18)—including science's 10 central methods and tools most used to spark those major discoveries. These top 10 innovations encompass Ernst Ruska's electron microscope (19 ), Kary Mullis's PCR method (20), Charles Townes's maser/laser (21), Felix Bloch and Edward Purcell's NMR spectrometer (22), Archer Martin and Richard Synge's partition chromatography (23), Arne Tiselius's electrophoresis (24), Ernest Lawrence's particle accelerator (25), Ronald Fisher's modern statistics (4), Theodor Svedberg's centrifuge (26), and Max von Laue's X-ray diffraction (6). Each of these tools sparked at least four later nobel–prize discoveries. We then analyze the data by assessing all 149 nobel–prize–winning method discoveries—including the top 10—compared with all other 384 nobel–prize discoveries (experimental and theoretical breakthroughs).

Scientific “methods” are defined as systematic techniques—such as controlled experimental, statistical, and computational methods; scientific “tools” are systematic instruments—such as telescopes, X-ray devices, and electron microscopes; both are used to study the world and extend our ability to observe, measure, and analyze and are general purpose. This means they can be applied to different questions and often domains. In practice, researchers often use the words method and tool interchangeably—and we do too for simplicity. We are not referring to cognitive abilities like observation and hypothesizing or theoretical frameworks. Major discoveries are defined as new experimental, methodological, or theoretical breakthroughs that mark an entirely new way to understand the world, open new paths of inquiry, and later have a proven, lasting impact on science. These breakthroughs range from the structure of DNA and gravitational waves to CRISPR gene editing and exoplanets. Details on other data collected for a given variable are provided when introduced.

## How science's powerful tools of discovery emerge

We begin by examining the 10 central methods and instruments most used to spark major scientific discoveries and the inventors behind them. Each of these top 10 transformative tools triggered multiple nobel–prize discoveries across fields, beyond the original fields they emerged in. But what drove these researchers to develop science's most transformative discovery tools and methods? What motivated them to rethink the methods they used to explore the world—and create entirely new ways of doing scientific research?

We find that Svedberg created the ultracentrifuge to overcome the limits of the ultramicroscope in studying particles (26). Tiselius invented electrophoresis that addressed constraints of the ultracentrifuge and the struggles of biochemists to separate proteins effectively (24). Martin and Synge designed partition chromatography to resolve the shortcomings of earlier chromatography methods (23). Each of these inventions unlocked new ways of separating and studying substances. Von Laue generated X-ray diffraction that enabled going beyond traditional X-ray analysis and probing the atomic and molecular structure of materials (6). Knoll and Ruska pioneered the electron microscope that broke the optical barrier of light microscopes by bypassing the diffraction limit of visible light that prevented visualizing tiny structures (19). Bloch and Purcell developed NMR spectroscopy that advanced earlier techniques, especially Rabi's molecular beam method, to uncover a powerful new way of probing atomic nuclei with unprecedented precision (22). Fisher invented modern statistical methods to tackle the inadequacies of earlier data analysis techniques, providing rigorous tools to design experiments and draw reliable conclusions (4). Townes created the maser that overcame limitations in generating and controlling electromagnetic waves and soon led to the laser—though he did not have a specific application for the tool in mind (21). Mullis invented the PCR method to make the process of DNA amplification and analysis much more efficient and rapid (20, 27). Lawrence constructed the particle accelerator that enabled vastly expanding existing models of linear accelerators (28).

This trend is striking across science: it is often applied researchers—not generally theorists—who spot a tool's defects, but rather than commonly accepting them, they seek to tackle them. It is generally technical bottlenecks—when methods fall short and progress hits a wall—where breakthroughs begin. The origins of science's best tools challenge a long-standing assumption—that theories play the central role in driving science; yet science's most transformative tools generally arise not from abstract theorizing but from practical deficits in earlier tools.

The overlooked flaws of tools are key in driving scientific progress. Scientific breakthroughs commonly begin not with just a grand question but with a technical obstacle impeding investigation. This means the gaps in our instruments can be just as crucial as the gaps in our knowledge. Because tools shape the questions and theories that we can even formulate and the discoveries we can make. By tracing their career paths and the key turning points in their work, we uncover six key factors behind science's greatest tool breakthroughs.

One, we find that the pioneering inventors of these top 10 general tools of discovery generally ran into methodological limits and were motivated by the need to create a new tool by pragmatically “overcoming a constraint to an existing tool.” Working within conventional scientific fields, they encountered barriers that hindered them in studying key phenomena using available tools. And they set out to fill these method gaps—with, for example, DNA analysis being slow and inefficient until Kary Mullis developed a simple way to rapidly amplify DNA that ultimately transformed genetics: the PCR method (20).

Two, these researchers each “shifted their research focus” from addressing scientific questions to tackling method and tool questions. They studied and worked in established fields like physics and chemistry; but transitioned from traditional science to an unconventional pursuit of extending our tools to explore the world in new ways—with, for example, Theodor Svedberg studying macromolecules and colloids but then turning to how to measure their properties more accurately, which led to inventing the ultracentrifuge (26).

Three, they took a very practical “experimental approach,” not simply theorizing about the limitations but tackling specific shortcomings of our tools that held back progress—with, for example, Ernst Ruska iteratively refining electron lenses until he had a working electron microscope with vastly improved resolution like nothing before (19).

Four, these innovators commonly produced the new tool “at low or modest costs” and not in large research teams. Motivated, resourceful researchers can make foundational breakthroughs with little resources and simple materials—from Mullis’ PCR method (20) and Tiselius’ electrophoresis (24) to Fisher's modern statistics (4).

Five, they were “primarily interdisciplinary” scientists, with eight out of 10 trained in different fields. This enabled them to generate new connections by often drawing on methods and evidence from different domains—with, for example, Ernest Lawrence's degrees in physics and chemistry enabling him to better understand the nature of atomic interactions and design the first practical particle accelerator (25).

Six, these inventors were “not prominent scientists before designing the groundbreaking tool.” Most were relatively unknown and did not make a major discovery before—with, for example, Mullis, an unknown biotech researcher, and Ruska, Tiselius, and Synge PhD students at the time (Table 1).

**Table 1:** How science's 10 central methods and tools were developed that triggered most nobel–prize discoveries.

Method or tool	Year developed	Inventor's name	Traits at the time of the discovery					Field of degree(s)	Won Nobel Prize for the tool	Cost to develop tool^a^	Country of birth	Fields using the tool	How the method or tool works—and its general use
			Education level	Age	No. of people making tool	University/top 50 ranked	Field of research						
PCR method	1985	Kary Mullis	PhD	41	1	Cetus (biotech company)/no	Chemistry	Biochemistry, chemistry	Yes, 1993	Low	USA	Medicine, genetics, biochemistry	Amplifies a small DNA sample (e.g. a drop of blood) quickly into millions of copies—and enables medical diagnostics (like detecting HIV) and paleontologic findings (identifying species using fossils) (20)
Maser/laser	1954	Charles Townes	Professor	39	1	Columbia University/yes	Physics	Physics, modern language	Yes, 1964	Medium	USA	Physics, computer science, astronomy, medicine	Produces concentrated beams of light or microwaves—and is used to probe molecular structures and deep space and applied in DNA sequencing, fiber optics, laser surgery, and computers (21)
Spectrometer, NMR^b^	1946	Felix Bloch and Edward Purcell	Professor	38	2	Stanford University/yes	Physics	Physics, electrical engineering	Yes, 1952	Medium	Switzerland/USA	Chemistry, physics, biology, medicine	Visualizes magnetic fields around an atomic nucleus—and is used to decode the structure of proteins, produce life-saving drugs, and observe tissue in our body (22)
Chromatography, partition	1941	Archer Martin and Richard Synge	PhD (research was part of PhD thesis in 1941)	29	2	Wool Industries Research Associate/no	Chemistry	Physics, chemistry, physiology, biochemistry	Yes, 1952	Low	UK	Chemistry, biology	Separates substances in mixtures and determines their composition—and enables identifying and understanding, e.g. amino acids and sugars (23)
Electron microscope	1933	Ernst Ruska	Engineering degree (research was part of PhD thesis in 1934)	26	1	Technical University of Berlin/no	Physics	Electrical engineering, physical sciences	Yes, 1986	Medium	Germany	Biology, medicine, physics	Magnifies minuscule objects using the wavelength of an electron—and enables studying microorganisms, nanoparticles, electrochemical reactions, crystals, and molecules (19)
Electrophoresis	1930	Arne Tiselius	PhD (research was part of PhD thesis in 1930)	28	1	Uppsala University/no	Chemistry	Chemistry	Yes, 1948	Low	Sweden	Chemistry, biology	Separates substances by moving charged particles in a fluid—and applies them to analyze, e.g. DNA and proteins, paternity tests, and DNA fingerprints (24)
Particle accelerator	1929	Ernest Lawrence	Professor	28	1	Berkeley, UC/yes	Physics	Physics, chemistry	Yes, 1939	Low (to high)	USA	Particle physics, nuclear medicine	Propels charged particles to high speeds using electromagnetic fields—and is used to study matter and proteins, develop new drugs, and create cancer therapies (25)
Modern statistics	1925	Ronald Fisher	Bachelors	35	1	Rothamsted (agricultural research institute)/no	Statistics and genetics	Astronomy (w/specialization in mathematics)	No	Low	UK	All scientific fields	Used to collect and analyze large datasets and run experiments—and applies them to examine almost any phenomenon, from diseases in populations to planets, societies, and even science itself (4)
Centrifuge	1924	Theodor Svedberg	Professor	40	1	Uppsala University/no	Chemistry	Chemistry	Yes, 1926	Medium	Sweden	Chemistry, biology, clinical medicine	Spins samples at ultrahigh speeds at over 20,000 revolutions a minute—and is used to separate small particles in fluids, gases, and liquids, like cells and viruses (26)
X-ray diffraction	1912	Max von Laue	PhD	33	1	University of Munich/no	Physics	Mathematics, physics	Yes, 1914	Low	Germany	Chemistry, physics, material science, biomedicine	Scatters X-rays using crystals—and applies them to uncover crystal structures, atomic structures of matter including proteins and identify cancers (6)
Average of 10	…	…	40% professors	34	1.2	30% at a top 50 university	…	80% interdisciplinary	90%	Low to medium	70% in Europe	…	…
Comparison group—all other nobel-prize discoveries	…	…	69% professors	39	1.4	38% at a top 50 university	…	54% interdisciplinary	100%	…	45% in Europe	…	…

The 10 central methods and tools are the most used to make the 533 nobel–prize discoveries. The comparison group (the last row) reflects all other 524 nobel–prize discoveries—with modern statistics not included, as it did not win a Nobel Prize. While the particle detector (1911) is at the threshold of making it into the top 10, the PCR method (1985) is included, as it illustrates a powerful, more recent tool discovery that also received a Nobel Prize and was used in triggering 10 major discoveries (20). The year reflects when the tool was first created, while all have been vastly improved.

^a^The cost to develop the tool is based on three categories: low under 1,000 US$, medium between 1,000 and 10,000 US$ and high above 10,000 US$, in 2025 prices. Developing the PCR method—in Mullis's words—required “no more than a test tube, a few simple reagents and a source of heat,” costing less than a few hundred US dollars (27). It cost Townes about 500 US$ to create the maser in 1954 (5,944 US$ in 2025 prices) (29). Building the first NMR spectrometer in 1946 cost Bloch 450 US$ (7,379 US$ in 2025 prices) (30). Partition chromatography was developed by Martin and Synge and required just water, filter paper, and a solvent—all cheap lab supplies (23). Constructing the first electron microscope cost Ruska about 500 Reichsmarks in 1933 (∼2,800 US$ in 2025 prices) (31). Tiselius developed electrophoresis at low cost, using a U-tube, a thermostat and a microphotometer (32). Creating the first operational particle accelerator prototype in 1929 cost Lawrence about 25 US$ (467 US$ in 2025 prices) (33), and later scaled-up accelerators about 2,500 US$ ($46,750 US$ in 2025 prices) (34). Modern statistics was developed by Fisher through his everyday work, using little more than a low-cost calculator (4). After Svedberg developed the centrifuge, he then obtained 25,000 Swedish crowns in 1924 to further expand his research (about 123,700 US$ in 2025 prices) (35). X-ray diffraction was developed by von Laue using an X-ray tube, a small crystal, and a photographic plate—all materials purchased at low costs (36).

^b^While the first spectrograph was invented in 1859, an entirely new form of spectroscopy based on magnetic resonance—namely NMR spectroscopy—has since become far more important.

**Figure 1:**
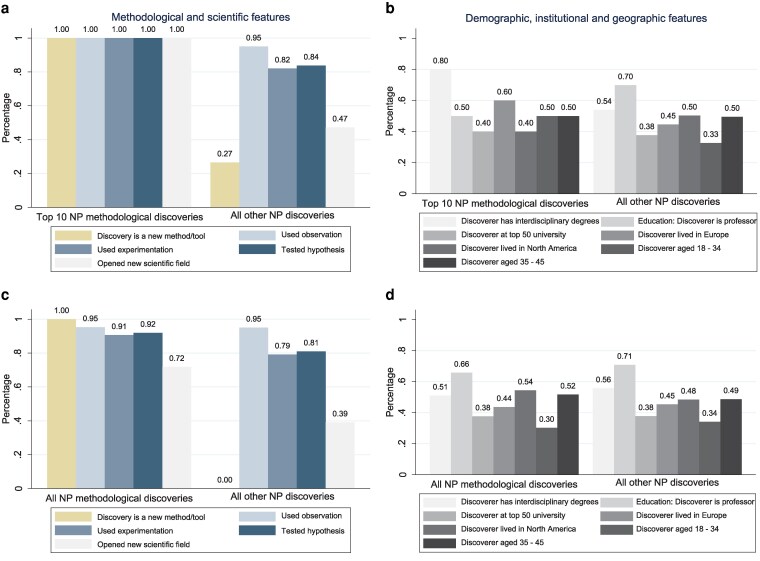
Nobel–prize–winning methods and tools compared with all other nobel–prize–winning discoveries, by features at the time of the discovery. The data reflect all 533 nobel–prize discoveries: with the top 10 nobel–prize–winning methods and tools compared with all other 523 nobel–prize discoveries (a and b), and with all 149 nobel–prize–winning methods and tools compared with all other 384 nobel–prize discoveries (c and d). NP stands for Nobel Prize. We outline the top 10 nobel–prize–winning methods and tools in Table 1, with the particle detector included here instead of modern statistics, as it did not win a Nobel—each of these tools has been applied to develop at least four later nobel–prize discoveries. Each of these discovery-making publications is categorized as relying on a new method or tool if it was recently developed and applied for the first time to the given problem (bar 1 in a and c), as using observation if the study describes collecting observational data (using eyesight) (bar 2), as using experimentation if the study ran an experiment (bar 3) and as testing a hypothesis if the study formulated and examined a proposed explanation (rather than doing exploratory research) (bar 4). They are categorized as opening a new field if the discipline they launched returns at least 25 results on Google Scholar by searching “field of *x*” (such as “field of chemistry” or “field of genetics”); and we verified that the scientists who opened the fields are described in scientific publications as the “founder,” “father,” or “mother” of the discipline (bar 5). For b and d, data on each discoverer's age at the time of discovery, their education level and interdisciplinary degrees, and where they lived are based on Encyclopaedia Britannica (37) and official Nobel Prize documents (18). Discoverer with interdisciplinary degrees is defined as having two or more degrees in different fields. All professors have a PhD. Discoverer at a top 50–ranked university is based on whether they were at such a university worldwide according to QS World University Rankings 2021 (38). Data are available as Dataset S1.

We next take a broader approach and compare the features of the top 10 nobel–prize–winning methods and tools—those sparking the greatest number of later nobel–prize discoveries—with the features of all other 523 nobel–prize discoveries across science. We find that these top 10 discovery tools were all developed through direct observation and experimentation, while other nobel–prize discoveries were less likely to rely on these approaches (Fig. 1a). Science's pioneering method-makers shared several unique and surprising traits: not only were eight out of the 10 interdisciplinary—each earning two or more degrees in different fields before their breakthrough but half of them were not professors and only completed a PhD or less and were under 35 (Fig. 1b).

We next take an even broader approach by examining all 149 nobel–prize–winning method discoveries—including the top 10—and compare them to all other 384 nobel–prize discoveries (experimental and theoretical breakthroughs). What stands out is that these groundbreaking methods and instruments were more likely to require experimenting and lead to a new field than other discoveries (Fig. 1c).

Exploring science's most influential toolmakers, we uncover a unique pattern—one that challenges a common assumption about where innovation happens and who drives it. We find that among these 10 great toolmakers, one group consists of “low-profile researchers” not at top universities, developing four of the 10 most powerful tools. A second group is made up of “academic outsiders”*—*researchers in tech and biotech companies that are highly applied research environments, inventing three of the 10 tools. This is surprising since 99% of all other nobel–prize discoverers were at universities or research institutions. And a third group involves “higher-profile researchers” at top 50 academic institutions, creating three of the 10 (Table 1). More broadly, exploring all 149 nobel–prize–winning method breakthroughs, we find a similar trend: almost two-thirds of these toolmakers were not based at the world's top 50 universities (Fig. 1d). Innovation usually begins not with privilege or prestige but with a technological gap.

These 10 pioneering methodologists were also younger: 34 years old on average at the time of their innovation, compared with 39 for all other nobel–prize discoverers. The most transformative tools that change the course of science can emerge before a career has even properly started—with three publishing their tool breakthrough as part of their PhD thesis. Leading toolmakers are thus often not elite researchers at the peak of their careers but rather typical scientists when looking at their background. This highlights that applied and resourceful researchers—regardless of reputation and institution—can turn their attention toward tackling practical method problems that can transform science. Another unique finding is that most of these 10 general tools were generated at low cost, requiring <1,000 US$ (in 2025 prices; Table 1).

Four common ways exist to develop new methods. One powerful way is “creating entirely new methods” not yet conceived—like chromatography for separating chemical substances. A second transformative way is “extending methods in novel ways” that represent a new method not ever used before—like gas chromatography that vastly upgraded chromatography. A third innovative way is “combining methods in novel ways” that introduces a new method not yet leveraged before—like X-ray crystallography that joins X-ray methods with crystal analysis (6). A fourth foundational way is “adopting new methods developed in other fields”—like NMR spectroscopy created in physics and then used for the first time ever in chemistry, medicine, and biology (22). In each of these cases, we triggered major discoveries with new methods never employed before to a given question.

New methods in AI, machine learning, and big data are also increasingly pioneered by tech companies for commercial use and then applied by scientists. These not only make science more efficient but also highlight an often symbiotic relationship between industry and science.

## Scientific table of methods: mapping and fusing methods and tools of discovery

Mendeleev transformed chemistry not by discovering a new element but by developing a new structure: the periodic table. He arranged the elements—by weight and chemical resemblance—so his students could better understand their properties. This remarkable new structure led to predicting and uncovering unknown elements. Similarly, science is organized to make it more accessible. Science textbooks categorize fields into neatly bounded disciplines—physics, chemistry, biology, psychology, and history—and present the scientific method as “the collection of data through observation and experiment, and the formulation and testing of hypotheses” (39). This traditional architecture is widely adopted across science. But is it a natural categorization of science? It is one among other ways to structure science. In fact, it can constrain us by overlooking two key features of how science actually works and progresses: first, the sophisticated methods and tools that do heavy lifting behind science's advances, and second, science's often interdisciplinary nature. So what if we restructure science not just around its subject matter or its abstract ideals but also around the practical methodological structure that drives science and unlocks discovery? Here, we tackle this question—developing a conceptual framework.

To begin, what are the most cognitively demanding methods and tools in science? At one end of the spectrum, we find complex “modeling and simulation methods”—techniques that demand elaborate formalization, iterative refinement, and greater abstraction. Whether modeling climate systems, biological processes, or economic behavior, these methods force us to make critical decisions that shape our findings: what should we simplify, exclude, and mathematically represent? “Mathematical methods” like calculus and algebra demand much theoretical reasoning, often far removed from everyday intuition—think of Newton's laws expressed using calculus (40). “Statistical inference methods,” too, are cognitively intense and involve navigating multiple layers of complexity. Moving from raw data to robust conclusions and predictions demands using methods like frequentist hypothesis testing, Bayesian inference, and regression analysis—all requiring reasoning about uncertainty and margin of error. These methods continuously evolve, with ongoing debates over *P*-values, effect sizes, and the replication crisis (41, 42). In turn, “quasi-experimental methods” are used when controlled experiments and randomization are not feasible. In fields like epidemiology and economics, researchers can at times rely on natural experiments or instrumental variable methods to approximate causal effects from real-world data (43). In contrast, “controlled experimental methods”—like randomized controlled trials—are built on clearer foundations but remain cognitively heavy, requiring careful technical judgment in isolating causal effects and managing bias (44).

“Sampling methods” are central in data-driven science and occupy a unique methodological middle ground. Take well-designed longitudinal studies that follow the same individuals over time—these demand meticulous planning to minimize problems of representativeness, attrition, and weighting the sample. We then move to “experimental tools”—such as centrifuges, PCR, and chromatography devices—that uncover data not directly accessible to our senses. Their output often later feeds into statistical analysis.

At the other end of the spectrum lie “indirect observational or measurement tools,” like X-ray crystallography and spectrometers. These instruments enable us to infer structures not directly observable like atoms and molecules—such as DNA's double helix. Their use can become streamlined and routine through standardized protocols. At the simplest conceptual level are “direct observational or measurement tools”—light microscopes, telescopes, thermometers etc.—that extend our sensory reach in intuitive ways resembling human senses. They do not demand deep abstraction. This continuum highlights a rich hierarchy of cognitive demands: from many methods—like complex experimental methods driven by elaborate statistics—demanding several years to master and complex decisions in each study, to some tools that require much less effort and minimal training to use—like light microscopes and thermometers. Many education systems reflect this gradient, introducing observational tools early and progressing toward more complex methods.

**Figure 2:**
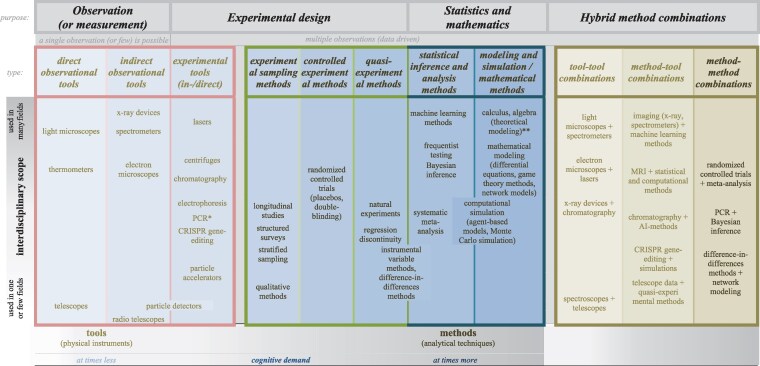
Scientific table of methods: a taxonomy of science's methods and tools—by type, purpose, interdisciplinary scope, and cognitive demand. This taxonomy is developed here based on the methods and tools used to spark science's major discoveries—and includes how broadly they are used across fields. Some tools and methods—like lasers and machine learning—can fall into different categories, with lasers, for example, applied as both an observational tool (laser interferometry in physics) and an experimental tool (surgical lasers in medicine). Experimental methods—like controlled trials—produce data that flow into statistical analysis, linking experimental design with statistical inference. *Some tools are often described as methods or techniques, such as PCR and CRISPR, and vice versa—though these are not methods in the abstract sense like statistical inference. **Some mathematical methods are used without relying directly on data but indirectly on previously collected data and findings. Methods can also be further classified by features like scalability, automation, reusability, precision, data type, and known limitations.

To better guide how we catalyze innovation across science, we develop here a taxonomy of science's methods and tools—a kind of “periodic table of methods.” Surprisingly, no framework yet exists that systematically maps methods and tools across science—from telescopes to t tests. By mapping the method landscape, the taxonomy reveals gaps and possibilities—helping guide where underused or unused techniques can be applied to catalyze discovery in unexpected domains. This science-wide classification can also offer researchers, educators, and innovators a structured way to think and rethink about the range of scientific methods and how we can link, adapt, and recombine them in innovative ways to generate new kinds of knowledge. We can classify—and compare—each method and tool as follows:

- Type and purpose: We differentiate between physical instruments (like microscopes) and analytical methods (like randomized controlled trials). We also categorize them by their function (observational, experimental, statistical etc.) and their subcategories (like direct or indirect observational tools).
- Interdisciplinary scope: We categorize how transferable methods are across domains—from field-specific impact (like radio telescopes) to interdisciplinary impact (like microscopes and PCR) and even (nearly) universal impact (like statistical and AI methods).
- Cognitive demand: We distinguish across the range of methods, with some techniques requiring increasing analytical effort, constant refinement, and much judgment (like complex statistical modeling), while some tools are increasingly standardized and intuitive across researchers (like light microscopes; Fig. 2).

Ultimately, a scientific table of discovery methods is a map of explored and unexplored method options that can help identify and predict untapped combinations of tools and where the next breakthroughs can come from. It captures several key features:

- Like elements in the periodic table, scientific methods and instruments can be thought of as foundational units.
- Just as we combine elements from the periodic table to create entirely new compounds, we can fuse a vast array of tools across our method landscape to create new compound methods that trigger breakthroughs.
- Just as there is no fixed number of chemical compounds we can form from over 100 known elements, there is no set limit to the number of powerful composite methods we can invent from science's developed methods and tools.
- We can bind, mix, and match two elements or methods, or three, four, or more.

Techniques like randomization, blinding, and/or placebos, for example, can be fused together to form stronger controlled experiments; their outputs then feed into statistical analysis; these in turn can be aggregated through meta-analysis—each methodological element linking together toward greater scientific rigor. The key insight can be powerful: “each fusion is a potential catalyst for discovery.” Many combinations are remarkably straightforward: most methods and tools generate data (imaging tools, PCR, surveys etc.) that can be linked to those that process and analyze data (statistical inference, machine learning) and to those that model behavior and systems (simulations and mathematical models), and so on.

The combinations are nearly endless—AI methods plus spectroscopy; MRI plus statistical and computational methods; lasers plus machine learning methods; the CRISPR method plus Bayesian inference or simulations (across populations or species); the PCR method plus network modeling (of how viruses spread); and so on. Exploring science's major discoveries, we can imagine countless fusions that hold high potential: combine chromatography with machine learning to predict new compound structures or optimize separations through learned molecular patterns. Link radio telescope data to quasi-experimental and causal inference methods to separate astronomical signals from noise and distortions. Connect centrifuges to AI imaging to train models to detect abnormalities in samples, and so on (Fig. 2).

## “Methods labs” and “methods hubs”: why we need to establish global incubators of innovation

Small, unexpected connections between method-curious researchers can prove important. Ernst Ruska, who created the groundbreaking electron microscope, had interactions with Max von Laue, the inventor of X-ray diffraction (6, 19). Kary Mullis designed the extraordinary PCR method while working at the biotech company Cetus whose co-founder was Donald Glaser, the pioneer behind the bubble chamber—a particle detector (20, 27). Remarkably, Arne Tiselius developed electrophoresis using the ultracentrifuge that his doctoral supervisor Theodor Svedberg recently designed, who allowed Tiselius to pursue his own independent research (24, 26). And later, Richard Synge—the co-inventor of partition chromatography—spent a year researching with Tiselius (23). These are not footnotes in the history of science—they are catalytic tools that each enabled dozens of discoveries.

These few exceptional inventors did not directly work together but what unites them is that they took place within the same environment that could inspire focusing on new techniques. These few researchers, with a methodological eye, deviated from conventional research paths—and in doing so, they reshaped the course of science through their new tools. Imagine what would happen if such extraordinary, unexpected interactions were not the rare exception, but systematically cultivated and the norm. There is an enormous untapped potential of triggering innovations in tools: we need to begin intentionally creating dedicated spaces within and beyond universities around the world where method-focused minds from different fields, backgrounds, and toolkits can converge to invent, merge, adapt, and share new methods—what we call here “methods labs” and “methods hubs.”

Just being embedded in a methods hub—surrounded by researchers designing the next generation of instruments—can inspire synergies and breakthrough innovations, even among researchers working independently. By examining science's major methods and discoveries, we reveal that Cambridge's Cavendish Lab stands out as the best and closest the world has come to a true methods lab. In just a few decades in the early 20th century, it produced a cascade of world-changing inventions. At the Cavendish Lab, Charles Wilson invented the iconic particle detector (cloud chamber) in 1911. Francis Aston constructed the mass spectrograph in 1919. Patrick Blackett developed the Wilson cloud chamber in 1932. John Cockcroft and Ernest Walton built an improved particle accelerator that same year. Pyotr Kapitsa devised a method to produce liquid helium at scale in 1934. And Martin Ryle designed an enormous radio telescope system in 1954. These innovations did not emerge by chance but were engineered in an exceptional method environment. J.J. Thomson, the nobel–prize–winning physicist and director of the Cavendish Lab, mentored Aston, whose mass spectrograph built on Thomson's earlier 1913 prototype (18).

Across the Atlantic, Bell Labs later served as another hotbed of innovation. In the span of about four decades, its researchers transformed key parts of science and technology: Shockley, Bardeen, and Brattain invented the groundbreaking transistor in 1947. Arthur Schawlow engineered Doppler-free spectroscopy in 1958. Jack Kilby pioneered the microchip in 1959. Willard Boyle and George Smith conceived the CCD sensor in 1970. Steven Chu developed Doppler cooling in 1985. And Arthur Ashkin devised optical tweezers in 1987.

Other smaller examples include Scripps Research in California, where Benjamin List invented an environmentally friendly tool for constructing molecules—organocatalysis—in 2000, and where Barry Sharpless developed click chemistry in 2001. And at Berkeley, Ernest Lawrence built the first particle accelerator in 1929, and Luis Alvarez created the hydrogen bubble chamber in 1959. All of these innovative scientists won a Nobel Prize for these breakthrough inventions (18).

The Cavendish Lab—peaking from the 1910s to the 1950s—and Bell Labs—from the 1940s to the 1980s—were not just two productive labs, they were the two most prolific methods incubators in the history of science. Remarkably, none of these institutions was founded with the goal of advancing scientific methods. Yet they became, for a period in scientific history, the world's leading methods powerhouses—without a systematic, science-wide understanding of how transformative tools speed up progress. These innovations made in the Cavendish Lab—a part of Cambridge's department of physics—and in the Bell Labs were all made by physicists—except for Jack Kilby, an electrical engineer. But the vision of a network of methods labs worldwide is far broader: it is not confined to physics or limited to any specific field but spans across science and connects cross-disciplinary toolmaking and infrastructure.

Today, no such methods powerhouses exist. The Cavendish Lab and Bell Labs are the nearest we have gotten but are rather field-specific exceptions of the past. Yet they made clear that the invention of better tools can be just as transformative as the theories they later uncover.

**Figure 3:**
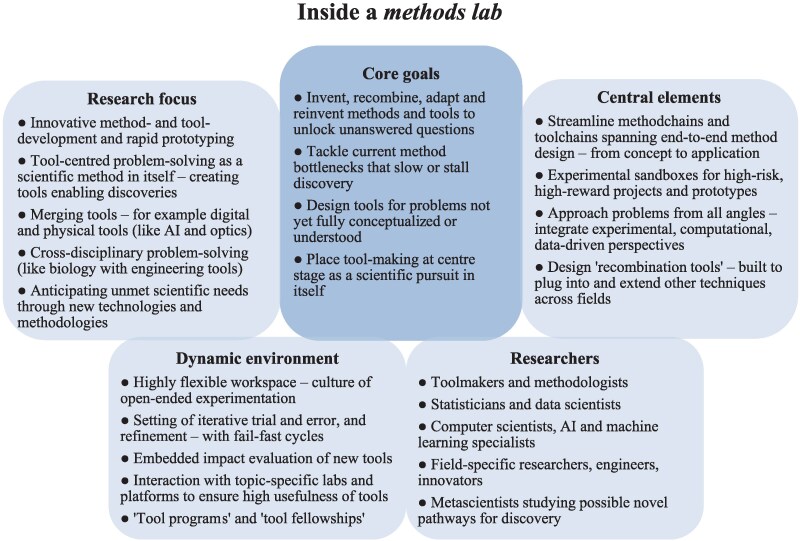
Creating “methods labs” as powerhouses of innovation and discovery. The concept of methods labs is developed here by synthesizing—and expanding on—patterns observed in the scientific methods and tools that have been created to trigger science's major discoveries.

The goal of methods labs would be to speed up the creation of cutting-edge tools by fostering a flexible environment of innovation—by merging, adapting, and testing methods and tools in new ways. Think of methods labs as “method accelerators” where researchers can freely explore hybrid approaches, prototypes, and experiment with new tools across disciplines more strategically and rapidly. These labs would deviate from traditional, disciplinary structures, where scientists are often constrained by existing methods and norms within their fields. These toolmaking labs would be where breakthroughs often begin—before the experiments and findings can even start. Here, we envision how such labs could be organized (Fig. 3).

Examining how the methods and tools behind science's major discoveries are developed, we observe that new transformative method innovations unlock new breakthroughs through five key stages:

- Method constraint: Generally, we run into a practical problem we cannot solve when using a current method or tool to study a phenomenon.
- Method scanning: Next, we need to scan our own field and related disciplines for a method or tool that can tackle that constraint (and if none exist, we go to the next stage).
- Method conceptualizing: At this point, we require conceiving a new method or tool designed to solve the constraint, expanding our cognitive or sensory scope.
- Method development: We need to then test, retest, and scale up prototypes of the new method or tool, enabling us to explore the world in fundamentally new ways.
- New method-driven discoveries and fields: Finally, we require applying the new method or tool to trigger new findings and advances.

**Figure 4:**
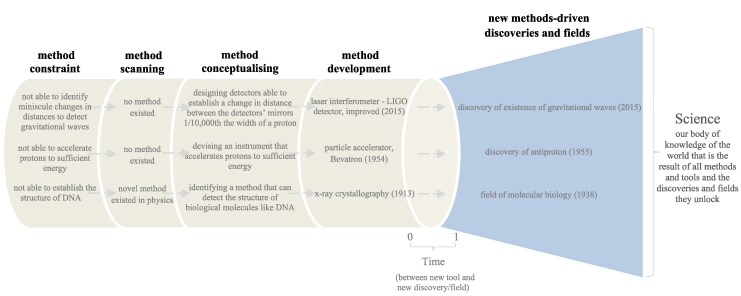
The five method-driven steps we take to trigger new discoveries and fields.

Take, for example, the extraordinary discovery of detecting gravitational waves in 2015. No tool was yet sensitive enough to reveal them. Researchers set out to develop an expanded massive laser interferometer, the LIGO detector. Uncovering the discovery that same year came down to an incredible methodological feat: they designed detectors thousands of kilometers apart that operate together with almost unimaginable precision—measuring a change in distance between the detectors’ mirrors 1/10,000th the width of a proton (Fig. 4). The high-precision instrument gave us a new window to the universe. Just 2 years later, the discovery earned a Nobel Prize—one of the fastest recognitions in Nobel history (45).

## Eight pathways to invent and reinvent our methods and tools—the making of discovery-triggering tools

We can begin to strategically plan and target ways to accelerate science: rather than waiting for opportunities to arise, we can actively develop new tools and adopt them from other fields. Innovation in methods follows two primary approaches. The most common strategy is to extend our tools, recombine them in novel ways, or invent entirely new tools. The other strategy is to scan other fields for tools to tap into to solve problems in one's own field. Yet because science still does not have the infrastructure set up to systematically foster tool-building (methods labs around the world) and rapidly spread knowledge about new tools across fields when they emerge, most breakthroughs continue to be made in an ad hoc way. There is more chance and less design in discoveries than necessary.

So what general steps increase our chances of breaking new ground? To answer this, we scan science's major discovery-making studies to identify patterns in how method innovations behind them came into being. We uncover eight key recurring pathways of how we create new methods and tools. We illustrate these general pathways using what is often seen as the leading method across the medical, behavioral, and economic sciences: randomized controlled trials (RCTs). This powerful experimental method randomly divides individuals into a treatment or control group to isolate and test the causal impact of, for example, a treatment, drug, or policy intervention. To date, more than 2 million RCTs have been conducted worldwide—on topics from vaccine safety to poverty alleviation policies (44). So what are the eight pathways we can take to design future breakthroughs?

One, “we can search other scientific domains for tools” that we can leverage and repurpose in our own field. Strikingly, the RCT method, first conceived in medicine in 1948, was only adopted decades later in neighboring disciplines, like economics and psychology (46, 47). When the method was finally imported in the early 2000s, it sparked a methodological revolution—shifting these fields away from looser observational correlations toward tighter causal precision (48). This method transplant forced entire disciplines to recalibrate their standards of evidence. Two, we can extend our tools by “combining new features” from tools “within the same scientific domain.” In RCTs, we link blinding techniques with placebos and other controls—design features that drastically reduce bias. Three, we can expand our tools by “combining new features” from tools “in other scientific domains.” In RCTs, we can randomize the entire sample of participants before the intervention even begins—a common technique in economics but not yet in medicine, although often feasible to ensure more reliable results (44).

Four, we can extend tools by “refining a feature.” In RCTs, we can adopt not only common double blinding (where participants and clinicians are unaware of who the treatment is assigned to) but also employ triple blinding or even “full” blinding. This ensures that all individuals involved in a study are blinded, including the data analysts and administrators (as each individual can unconsciously influence and bias results) (44 ). Five, we can expand tools by “developing an entirely new feature.” Rather than testing just one intervention at a time, RCTs can compare multiple treatments side by side. Imagine a trial comparing increased exercise, improved diet, not smoking, and a medical treatment simultaneously. This allows researchers to evaluate not just whether interventions work but reveals which combinations are most effective in improving our lives. Six, we can “invent a completely new tool” through major innovations (at times in pathways 2 to 5). Remarkably, the RCT method itself is such an invention: an extraordinary convergence of design features into a powerful, aggregate methodology (46). Imagine the AI-driven RCTs we can conduct: patients could be randomly assigned, for example, to either receive a diagnosis from a traditional physician using conventional tools or from a physician using an AI-assisted diagnostic system—trained to detect likely disease patterns using data from thousands of similar cases. The trial would not just test the treatment, but it would also test the tools of diagnosis themselves.

Seven, we can critically identify “methodological constraints, assumptions, and biases” of our tools—and then design strategies to reduce them. These strategies in RCTs include testing a new treatment against both a placebo and a conventional treatment to improve the relevance of the findings. They involve fully reporting both the treatment's positive and negative effects and clearly assessing how generalizable the findings are. Eight, we can detect “cognitive, sensory, and social constraints” and biases we face—and then devise new techniques to minimize them. In RCTs, we apply experimental controls, randomization, intention-to-treat analysis, and blinded peer review—each helps tackle bias (44, 49). Ultimately, through continual methodological innovations, the RCT method has become one of science's most remarkable methods for improving our health.

**Figure 5:**
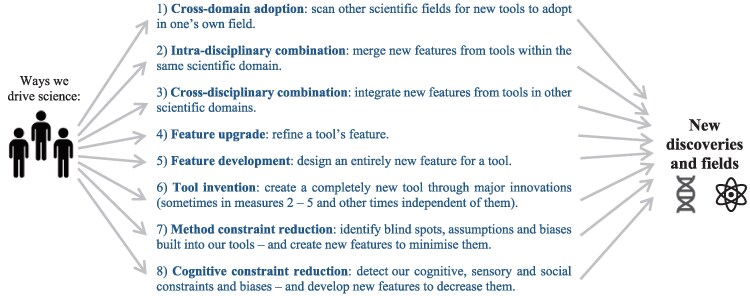
Practical guidelines for how we extend our methods and tools: eight pathways. The eight pathways were identified by screening science's major discoveries—and they can overlap in practice. For pathway six—tool creation—we commonly invent a new tool with an objective in mind. But in exceptional cases, tools are built with no direct aim or necessity at first, created as ends in themselves. The maser is such an example, but this “purpose-free” tool soon found wide application in science.

These strategies reveal a powerful insight: method innovation is not a rare cognitive moment; it is a systematic process. When we treat method-making as a central research goal, we can actively design discovery itself. There are countless opportunities to stretch and upgrade nearly all existing tools through vast combinations of features across disciplines. The sheer number of unexplored method configurations is enormous—especially when we think of the vast range of techniques across the experimental, statistical, and computational sciences. Any path we take requires shifting our focus to leveraging tools in new ways that open new avenues of exploration (Fig. 5).

These eight practical strategies are not just a map of how past discoveries have been sparked—they serve as a roadmap for future breakthroughs. Young students or established researchers can use these strategies to drive innovation in their work. Gaps in our tools do not just stifle progress; we find that they are its core inspiration. Because the limits of our tools largely determine what we can achieve experimentally and theoretically, it is crucial to tackle our tool constraints—and do so more efficiently. There is vast potential here: if researchers begin adopting this mindset, toolmaking may emerge as the most powerful meta-method in science and discovery. It would shift how we view scientific innovation: from sudden to systematic inspiration—from intuitive to intentional design.

Yet some new tools can face some resistance. Some encounter ethical constraints, others involve much technical complexity, and still others are entangled in economic or political hurdles. A striking example is CRISPR, the extraordinary gene-editing method that some scientists are hesitant to adopt for certain purposes—raising ethical fears over possible “designer babies” and irreversible ecological impacts (50). Other powerful tools demand greater training that can affect the pace of discoveries. Advanced computational methods and super-resolution microscopy promise greater efficiency, but their complexity can slow their adoption. Tools are transformative when more than a handful of scientists can use them. Some tools can be further influenced by commercial interests. New imaging instruments, CRISPR, and other tools tied to patents or licensed by private companies can limit who can leverage them. Still other tools depend on cross-disciplinary collaborations, like machine learning and AI methods that bridge the worlds of computer scientists and statisticians with biologists, chemists, and climate scientists. Scientists need to think of themselves not just as problem solvers but as method architects—designers of the very tools that shape their ability to know. Take also the remarkable AI-program AlphaFold, which required extensive interdisciplinary collaboration and transformed our ability to predict protein structures in biology and biochemistry with unprecedented accuracy. It has also accelerated research in drug discovery and genomics (18, 51, 52).

And of course, while scientific discoveries and tools have vastly benefitted our lives, some have unintended risks—like contributing to environmental degradation and climate change. Others also raise fears about some unforeseeable consequences of using AI tools in science. These may contribute to automation and job loss—not just in manufacturing, but in research and education—and to some feeling more socially isolated as machines can replace human interaction and even human judgment. Regulation and ethical oversight are essential—to ensure tools serve the public good (52).

It goes without saying that designing methods—like doing science itself—is often a process of trial and error. Many initial methods, when first developed, may not be useful enough or too limited in scope to survive and spur advances. Just like experimental results and theories, we only see methods that make it into publications.

So when are new methods most useful and have the biggest impact? It is when they solve a critical methodological bottleneck—visualizing or measuring something we could not before. When they significantly improve the efficiency or precision of our current methods, unlocking completely new perspectives, questions, and even entire research areas. And when we can apply and scale them across disciplines, catalyzing insights at the intersection of fields. New machine learning methods, electron microscopes, advanced statistical methods, etc. meet all of these conditions.

## Future directions and conclusion

Science has lacked a general understanding of how we create new tools. Without it, researchers until now have had to experiment and work out on their own—researcher by researcher—how to trigger a breakthrough and which new tools we need. Much time is lost, researchers continue in stagnated fields, and breakthroughs go unrealized just because the current system did not yet have a roadmap for building better tools. Relying on familiar methods simply because they are familiar is the reason why much research does not advance science.

When we invent, upgrade, combine, or adopt a new method or tool, we can predict where discoveries can come from next and when. It is about tracking the speed and direction of method innovations: this enables us to spot strong signals that discovery is soon to follow. The signal can come from a recently invented computational method, an upgraded particle accelerator, an advanced spectrometer combined with AI tools, or a new experimental method adopted from another field. Each helps us anticipate where the frontiers of science can soon move. In short, if a major new method or tool is developed, then the likelihood of new discoveries rises sharply.

Science has traditionally taken an indirect route to discovery: researchers have explored scientific questions that we often did not quite yet have the methods and technologies to answer. But imagine what would happen when we flip the research direction: when researchers pursue method questions first, designing and selecting new tools not just to answer today's questions, but to unlock tomorrow's. Our tools should no longer be seen as just means to do research but as active sources of innovation and inspiration that continually open new questions.

We can describe the era before a method revolution as when we commonly made advances in methods and tools in a surprisingly ad hoc and unplanned way with large time lags between new methods and discoveries. A method revolution would instead be marked by designing and developing methods and tools in a planned, strategic, and targeted way. Yet we have not yet systematically coordinated and fast-tracked tool development across science. Advances in tools would then move from the background to the foreground in understanding how we drive scientific advances. We can then begin actively engineering the conditions for making breakthroughs (Table S1).

Future scientists may view the advent of a methods revolution as the moment at which the entire scientific community became self-aware about how it progresses. It is the point at which we began to deliberately refine, combine, restructure, and invent methods and instruments that accelerate breakthroughs. The beginning of the revolution would be the moment where we collectively realize the fact that methods and tools are one of the most powerful frontiers we have yet fully explored. No more would our tools be studied by a small group of methodologically interested researchers who diverged from their initial training. A tool revolution in science would be driven by a new understanding across scientists: tools are not neutral, and advancing our tools changes the very kinds of questions we can ask and answers we can imagine. As the periodic table transformed chemistry by mapping its fundamental building blocks, science could gain from an evolving periodic table of methods: a living map of the methods, tools, and their vast recombinations that power discoveries across domains.

Overall, we propose seven key reforms to accelerate discovery and foster tool development: shifting a much larger share of researchers dedicated to tackling method bottlenecks; making the testing and expanding of our best methods a regular part of research; reconceiving science around method-driven research that is exploratory—not just question-driven; equally incentivizing method innovations among journals, prizes, universities, and funding bodies; developing new Methods Labs and Departments of Methodology—as incubators of innovation; shifting our main measure of success from just outputs (article citations) to focus equally on inputs—method innovations; and integrating deep methodological training into university degrees across science. A series of companion studies examine other aspects of science's major discoveries, including how discoveries emerge, the role of serendipity, the traits of discoverers, the cumulative nature of science, the scientific method, and the emergence of new fields (53–58)—and are highlighted in a larger book, “The Engine of Scientific Discovery” (59).

Ultimately, until we place our powerful toolbox at the center of science, we will continue to think of scientific discoveries as the most important feature of science—and not also the incredible tools that make them possible. We will keep viewing scientific discoveries as just being made by brilliant discoverers—and not also the brilliant tool inventors who enable triggering them.

## Supplementary Material

pgag107_Supplementary_Data
