# Supplementary material for: Discovery tools: How powerful new scientific methods and instruments emerge and catalyze innovation
Source: PNAS Nexus. 2026 Apr 9;5(4):pgag107. doi: 10.1093/pnasnexus/pgag107 (PMC13108597; doi:10.1093/pnasnexus/pgag107)
Supplement: pgag107_Supplementary_Data [file pgag107_supplementary_data.zip › PNASNEXUS-PNASNEXUS-2025-01208R-s02.pdf]

Discovery tools:
How powerful new scientific methods and instruments emerge and catalyse innovation

Supplementary material

Exploring science's top ten most used discovery tools, what do these instruments let us do—and what barriers have they broken through? And crucially, what limits currently hold them back that we need to tackle to spur future discoveries? Each major tool extends what we can know, but also faces limits that define the edge of what we can know (Table S1).

From supercomputers that struggle with processing power limits when modelling earth's complex climate, to MRI scanners that struggle with blurred motion in living patients, our tools all hit walls. Everywhere we look we find these bottlenecks waiting to be tackled, in each method we use—unlocking the next unexpected findings. Often the most important question we can ask ourselves to break new ground is: how can we better spot and tackle the bottlenecks facing our own tools? And the bottlenecks facing science's most influential discovery tools? That is the heart of much of how we push the current edges of science: not just running experiments and coming up with theories to explain what our existing tools reveal, but building the next generation of tools that let us see and imagine in entirely new ways.

Table S1. Developing new methods and tools drives science by reducing our constraints: the ten central tools used most to trigger discoveries

Method or tool	Year first developed	Human constraints (that the tool reduces)	What the tool enables us to study or explain (that would be impossible without it)	Current constraints of the tool (that we need to overcome to push our scientific frontiers further)
Laser	1960	Our inability to produce coherent light, make ultrafast measurements of distance and speed, and rapidly transmit large volumes of information	Study ultrafast interactions in matter, molecular structures, phenomena in deep space, and measure ultrashort time scales; develop fibre optics and laser surgery	Limits in power, efficiency and brightness of high-power diode lasers, and thermal constraints in solid-state lasers. [11]
Electron microscope	1933	Our limited vision to magnify and perceive miniscule objects using visible light, and the constraints of optical microscopes and limited resolution	Observe nanoparticles, electrochemical reactions, microorganisms, crystals and molecules—by using the shorter wavelength of electrons	Resolution limitations, challenges in observing living cells, sample size restrictions, and damage to samples from the electron beam. [60]
Chromatography	1931	Our constraints in separating and analysing chemical substances in a mixture	Analyse simple or complex mixtures containing vitamins, proteins and amino acids; isolate and purify natural compounds	No universal detector in high-performance liquid chromatography, [61] low detection limits at high resolution, difficulties with substances not volatile enough to separate. [62]
Electrophoresis	1930	Our limited capacity to separate and identify molecules by size and electrical charge	Separate and analyse biological molecules like DNA, RNA and proteins—vital for biomedical research and forensic science	Challenges in analysing highly complex mixtures; heating blurs the resolution, and limited capacity for large-scale applications. [63]
Statistics (modern)	1925	Our limited mental capacity to process large volumes of data, and complex relationships between variables	Study millions of data points across all fields, and more complex phenomena—from disease dynamics in populations and astronomical objects, to cells and the global economy	Constraints in sample size, scale and power; limited to studying phenomena we can represent statistically, constraints in the complexity of what we can model, and potential measurement error and sampling bias. [44]
Centrifuge	1924	Our inability to spin samples at ultrahigh speeds (greater than 20,000 rpm) to separate particles by density	Separate small biological particles like cells, viruses and nucleic acids from fluids	Limits on the volume of material we can process, [64] risks of cross-contamination between separated parts [65] and damage to samples from high-speed rotation. [66]
X-ray diffraction/ crystallography	1912	Our limited capacity to visualise the atomic structures of matter because of short wavelengths	Analyse the atomic and molecular structure of matter like proteins and nucleic acids; and detect bone fractures, pneumonia and certain cancers	Low intensity of x-rays for light atoms, difficulties to analyse crystal growth in real-time; [67] and overlapping signals in some diffraction patterns (x-ray powder diffraction). [7]
Spectrograph/ spectrometer	1859	Our limited senses to detect and analyse electromagnetic radiation across wavelengths beyond the visible spectrum	Study structures of atoms and molecules, determine the chemical makeup of planets and stars, and electron arrangements of elements in different energy states	Low sensitivity such as in NMR; [68] often require coupling with methods like chromatography to probe complex samples (as in mass spectrometers); and face computational limits in interpreting spectral data. [69]
Thermometer	1714	Our limitations in precisely perceiving and measuring variations in hot and cold temperature	Measure climate temperatures, surface temperatures in natural and industrial environments, and body temperature in health	Constraints in accurately measuring extreme temperatures, especially below -100°C and above +300°C, and issues of calibration and sensitivity. [70]
Telescope	1608	Our limited vision to magnify and perceive distant objects, detect different regions of the electromagnetic spectrum	Observe exoplanets, stars, sunspots, galaxies, nebulae and other astronomical objects across multiple wavelengths	Blurred resolution from the Earth's surface caused by atmospheric disturbance; and space telescopes also face size and resolution limits. [3]

Reducing such constraints can enable us to explore new phenomena and trigger new advances

These are the ten methods and tools most often used to make science's major discoveries. The year marks when the method or tool was first developed—though all have been vastly improved since. The final column highlights some of their current limitations that need to be tackled.
